# Plasma p-tau181 accurately predicts Alzheimer’s disease pathology at least 8 years prior to post-mortem and improves the clinical characterisation of cognitive decline

**DOI:** 10.1007/s00401-020-02195-x

**Published:** 2020-07-27

**Authors:** Juan Lantero Rodriguez, Thomas K. Karikari, Marc Suárez-Calvet, Claire Troakes, Andrew King, Andreja Emersic, Dag Aarsland, Abdul Hye, Henrik Zetterberg, Kaj Blennow, Nicholas J. Ashton

**Affiliations:** 1 Department of Psychiatry and Neurochemistry, Institute of Neuroscience and Physiology, The Sahlgrenska Academy, University of Gothenburg, Mölndal, Sweden; 2 Barcelonaβeta Brain Research Center (BBRC), Pasqual Maragall Foundation, Barcelona, Spain; 3 IMIM (Hospital del Mar Medical Research Institute), Barcelona, Spain; 4 Centro de Investigación Biomédica en Red de Fragilidad y Envejecimiento Saludable (CIBERFES), Madrid, Spain; 5 Servei de Neurologia, Hospital del Mar, Barcelona, Spain; 6 Department of Basic and Clinical Neuroscience, Institute of Psychiatry, Psychology and Neuroscience, King’s College London, London, UK; 7 Department of Neurology, University Medical Centre Ljubljana, Ljubljana, Slovenia; 8 Department of Old Age Psychiatry, Institute of Psychiatry, Psychology and Neuroscience, King’s College London, London, UK; 9 NIHR Biomedical Research Centre for Mental Health and Biomedical Research Unit for Dementia at South London and Maudsley NHS Foundation, London, UK; 10 Centre for Age-Related Medicine, Stavanger University Hospital, Stavanger, Norway; 11 Clinical Neurochemistry Laboratory, Sahlgrenska University Hospital, Mölndal, Sweden; 12 Department of Neurodegenerative Disease, UCL Institute of Neurology, London, UK; 13 UK Dementia Research Institute at UCL, London, UK; 14 Department of Psychiatry and Neurochemistry, Wallenberg Centre for Molecular and Translational Medicine, Institute of Neuroscience and Physiology, The Sahlgrenska Academy at the University of Gothenburg, Gothenburg, Sweden

**Keywords:** Alzheimer’s disease, Neuropathology, Braak, Blood biomarkers, p-tau181

## Abstract

The neuropathological confirmation of amyloid-β (Aβ) plaques and tau neurofibrillary tangles (NFT) remains the gold standard for a definitive diagnosis of Alzheimer’s disease (AD). Nowadays, the in vivo diagnosis of AD is greatly aided by both cerebrospinal fluid (CSF) and positron emission tomography (PET) biomarkers. Although highly accurate, their broad implementation is restricted by high cost, limited accessibility and invasiveness. We recently developed a high-performance, ultrasensitive immunoassay for the quantification of tau phosphorylated at threonine-181 (p-tau181) in plasma, which identifies AD pathophysiology with high accuracy. However, it remains unclear whether plasma p-tau181, measured years before the death, can predict the eventual neuropathological confirmation of AD, and successfully discriminates AD from non-AD dementia pathologies. We studied a unique cohort of 115 individuals with longitudinal blood collections with clinical evaluation at 8, 4 and 2 years prior to neuropathological assessment at death. The results demonstrate that plasma p-tau181 associates better with AD neuropathology and Braak staging than a clinical diagnosis 8 years before post-mortem. Moreover, while all patients had a diagnosis of AD dementia during life, plasma p-tau181 proved to discriminate AD from non-AD pathologies with high accuracy (AUC = 97.4%, 95% CI = 94.1–100%) even 8 years before death. Additionally, the longitudinal trajectory of plasma p-tau181 was assessed in all patients. We found that the main increases in plasma p-tau181 occurred between 8 and 4 years prior to death in patients with AD neuropathology and later plateauing. In contrast, non-AD pathologies and controls exhibited minor, albeit significant, increases in p-tau181 up until death. Overall, our study demonstrates that plasma p-tau181 is highly predictive and specific of AD neuropathology years before post-mortem examination. These data add further support for the use of plasma p-tau181 to aid clinical management in primary care and recruitment for clinical trials.

**Electronic supplementary material:**

The online version of this article (10.1007/s00401-020-02195-x) contains supplementary material, which is available to authorized users.

## Introduction

Alzheimer’s disease (AD) is the most prevalent cause of dementia, accounting for 50–60% of the 50 million reported cases worldwide, which is expected to triple by 2050 [25]. A definitive diagnosis of AD remains to be only possible via neuropathological examination that demonstrates the presence of the classical disease hallmarks, namely amyloid-β (Aβ) plaques together with tau neurofibrillary tangles (NFT) [6, 14]. However, increasingly, clinical assessment of AD is now being aided by neuropathologically validated biomarkers that reflect Aβ and tau pathologies which have led to the improved accuracy in diagnosing AD during life [9, 13, 20]. The importance of biological markers has been emphasised in the recent National Institute of Aging and Alzheimer Association (NIA-AA) Research Framework [13]. In this framework, AD is defined as a biological construct, documented by post-mortem examination or in vivo by biomarkers, and not as a clinical syndrome. Therefore, the term AD is applied whenever there is biomarker evidence of Aβ and tau pathology. There are two main types of biomarkers for AD, that is, neuroimaging and fluid biomarkers. Neuroimaging biomarkers include in vivo positron emission tomography (PET) using ligands specific for fibrillar Aβ [3] and paired-helical filament tau [17, 29]. Regarding the fluid biomarkers, the triad of cerebrospinal fluid (CSF) biomarkers, broadly referred as “*core AD biomarkers*”, are widely used in both clinical and research settings. They comprise Aβ42 (or the Aβ 42/40 ratio), phosphorylated tau181 (p-tau181) and total tau (t-tau), which reflect Aβ pathology, tau pathology and neuronal injury, respectively [21].

Despite their high specificity and sensitivity in detecting AD pathophysiology, both CSF and imaging biomarkers present certain limitations, e.g. perceived invasiveness or complexity attached to a lumbar puncture or limited access to and high costs for molecular imaging, which restrict the use of these biomarkers to specialised centres [21]. Therefore, a blood biomarker that reliably reflects cerebral Aβ and tau pathologies has huge potential as a scalable test for primary care and frequent disease monitoring in clinical and therapeutic settings. In recent years, numerous promising studies have explored the potential of blood biomarkers to provide information on cerebral pathology. Mass spectrometry [22, 28] and automated immunoassays [23] measuring Aβ species have proven highly accurate,however, the considerable peripheral expression of Aβ remains to be a significant cofounder for these assays, making the fold change in Aβ42/40 ratio in amyloid PET-positive individuals much less pronounced in plasma than in CSF [28]. On the other hand, blood immunoassays targeting tau species, specifically tau fragments phosphorylated at threonine 181, have shown promising results, proving to be reliable tools for AD diagnosis and correlating well with in vivo assessments of Aβ and tau pathologies [15, 16, 32].

To our knowledge, and despite the very promising results in blood p-tau181, all studies conducted so far have mainly focused on research cohorts accurately characterised by CSF or PET biomarkers. Some of these studies have also validated their results in a subset of pathologically confirmed cases [32] but it is unclear if plasma p-tau181, determined years before death, can predict the eventual neuropathological diagnosis of AD. Therefore, the main aim of this study was to investigate (1) if plasma p-tau181 specifically reflects AD pathology in neuropathologically confirmed cases, (2) if plasma p-tau181 would inform on a more accurate diagnosis of AD and highlight dementia of a non-AD type at the time of clinical assessment, and (3) if the longitudinal trajectories of plasma p-tau181 are different between neuropathologically confirmed AD patients, non-AD patients and controls. For this purpose, we measured plasma p-tau181 in a longitudinal cohort comprising cognitively unimpaired controls and participants with the clinical diagnosis of mild cognitive impairment (MCI) and AD dementia. At post-mortem, each patient was re-classified into control, AD and non-AD dementia based on a detailed neuropathological assessment.

## Materials and methods

### Study participants and design

**Fig. 1:**
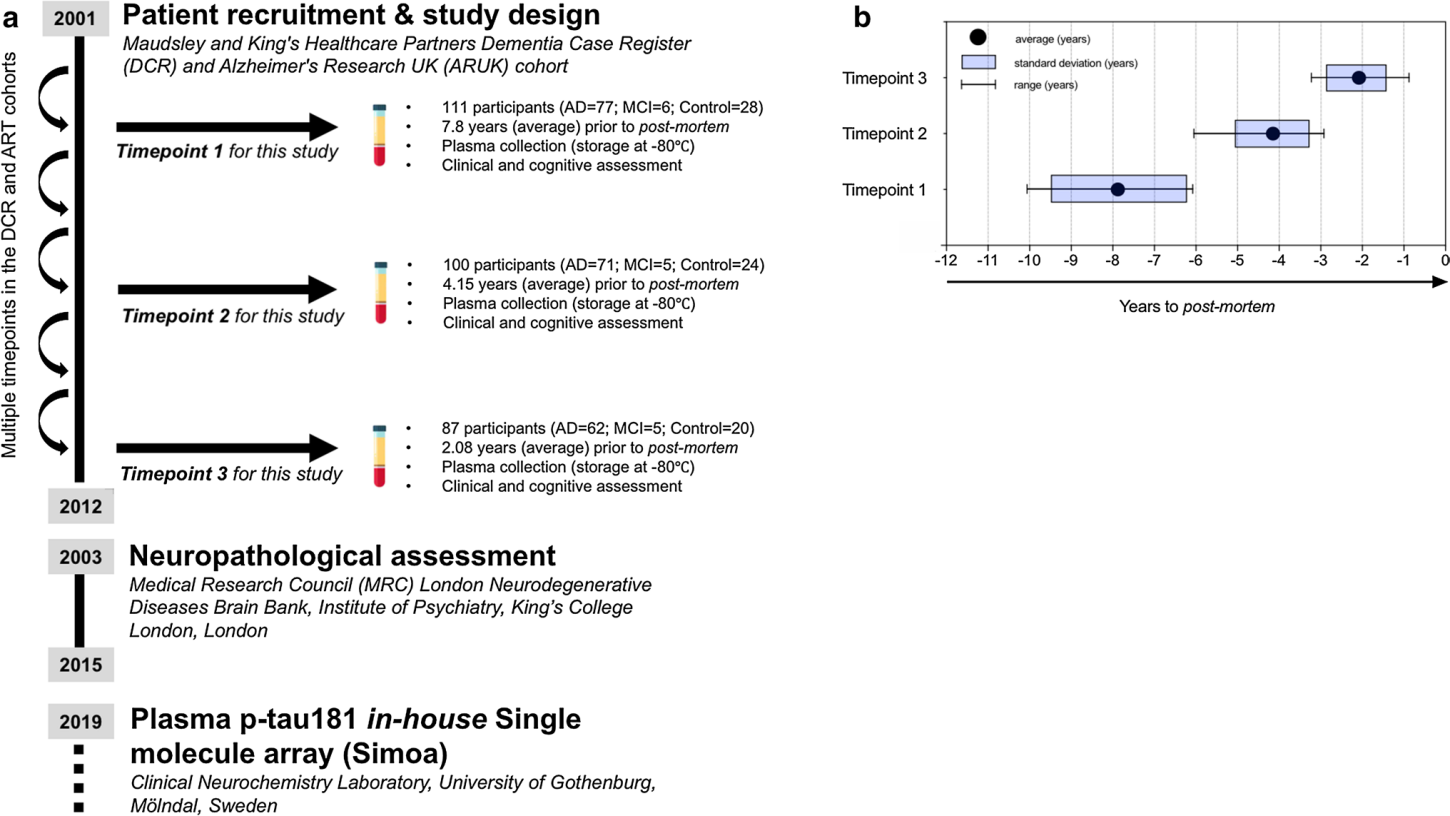
Study design. **a** The study design and timeline of sample collection, subsequent brain donation and plasma p-tau181 measurements. The current study included a total of 115 individuals from the Maudsley and King’s Healthcare Partners Dementia Case Register (DCR) and Alzheimer's Research UK (ARUK) cohorts, which underwent multiple clinical examinations between 2001 and 2012. To be included in the present study, individuals must have completed > 1 blood and clinical assessment via the DCR cohort and separately, a neuropathological assessment from the Medical Research Council (MRC) London Neurodegenerative Diseases Brain. Stored plasma samples were analysed for p-tau181 at clinical neurochemistry laboratory, Sweden. Plasma timepoints were specifically selected from each individual based on the time (in years) from the recorded date of post-mortem (**b**); timepoint 1 = 7.88 mean years (SD = 1.15, range = 6.33–9.43), timepoint 2 = 4.15 mean years (SD = 0.91, range = 2.90–6.05) and timepoint 3 = 2.08 mean years (SD = 0.70, range = 0.86–3.21)

The current study included 115 individuals selected from the Maudsley and King’s Healthcare Partners Dementia Case Register (DCR) [12], which incorporates the Alzheimer's Research UK (ARUK) cohort [11]. To be included in the present study, individuals must have completed ≥ 1 blood and clinical assessment via the DCR biomarker program and subsequently received a post-mortem neuropathological assessment through brain donation to the Medical Research Council (MRC) London Neurodegenerative Diseases Brain Bank, Institute of Psychiatry, King’s College London (which includes donation via the Brains for Dementia Research (BDR) program) (Fig. 1a). The clinical data and plasma samples from DCR were matched to post-mortem records by the co-authors and are an extension of our previously reported cohort [2].

Plasma collections or “*timepoints*” were specifically selected on the basis of time (in years) to the recorded date of post-mortem (Fig. 1b); timepoint 1 = 7.88 years (SD = 1.15, range = 6.33–9.43), timepoint 2 = 4.15 years (SD = 0.91, range = 2.90–6.05) and timepoint 3 = 2.08 (SD = 0.70, range = 0.86–3.21). Of the 115 patients included, a total of 83 individuals had plasma for all three timepoints, 17 individuals had two timepoints and 15 individuals with only one timepoint were available. At each timepoint, the clinical diagnosis of probable AD (AD dementia) was made according to Diagnostic and Statistical Manual for Mental Diagnosis, fourth edition and National Institute of Neurological, Communicative Disorders and Stroke–Alzheimer’s disease and Related Disorders Association (NINCDS–ADRDA) clinical criteria. Mild cognitive impairment (MCI) was defined according to Petersen criteria [12]. Standardized clinical assessments included the informant interview for diagnosis and the Mini-Mental State Examination (MMSE). Cognitively unimpaired (CU) individuals were either spouses of cases or recruited though primary care registers, all had MMSE > 26. The human biological samples were sourced ethically, and their research use was in accordance with the terms of the informed consent.

### Plasma p-tau181 measurement by single molecule array (Simoa)

All participants from the DCR were required to fast for at least 2 h prior to medical assessment. Venous blood was drawn and collected into sodium ethylenediaminetetraacetic acid (EDTA)-containing tubes. Within 2 h of collection, samples were centrifuged (8 min at 2000 × *g* at 4 °C) and stored at − 80 °C. Plasma p-tau181 concentration was measured using an ultrasensitive in-house single molecule array (Simoa) assay developed at the Clinical Neurochemistry Laboratory, Department of Psychiatry and Neurochemistry, University of Gothenburg, Sweden [16], on the HD-X platform (Quanterix, Billerica, MA, USA). In brief, the plasma p-tau181 Simoa assay is comprised of paramagnetic beads coupled with a mouse monoclonal capture antibody specifically targeting phosphorylated threonine 181 (AT270, Invitrogen) and biotinylated mouse monoclonal detector antibody directed against the N-terminal region of tau (Tau12, BioLegend). Full-length recombinant tau-441 phosphorylated in vitro by glycogen synthase kinase 3β (#TO8-50FN, SignalChem) was used as the calibrator. Further details about the assay and validation performance have been previously described [16]. Immediately before analysis, plasma samples were thawed, vortexed (2000 rpm) and centrifuged (10 min at 4000 × *g* at RT) and then diluted twofold with Tau2.0 buffer (Quanterix, Billerica, MA, USA). Plasma samples were randomised and analysed using identical batches of reagents. Plasma p-tau181 data were collected over three analytical runs, and all samples measured above the lower limit of quantification set for the assay (1.0 pg/mL). As a measure of assay precision, two quality control (QC) plasma samples were analysed in duplicate at the start and end of each run. The within- and between-run coefficients of variation for both QC samples were < 10%.

### Neuropathological diagnosis

Consent for autopsy, neuropathological assessment and research was obtained for all cases and the study was carried out under the ethical approval of the tissue bank. Block taking and neuropathological assessment were performed according to the standard criteria for the diagnosis of neurodegenerative disease. Assessment included Braak staging for NFT [7] and reporting of co-existing pathology such as cerebrovascular lesions, TAR DNA-binding protein 43 (TDP-43), and Lewy body pathology. Control cases were defined as showing no more than Braak stage II, age-related pathology.

### Statistical analysis

SPSS (IBM, Armonk, NY) and the R programming language (version 3.4.3) were used for statistical analysis and Graph Pad PRISM for visualisation. Associations between continuous variables were tested with Spearman’s rank-order correlation. Group differences were assessed with Mann–Whitney test or one-way Kruskal–Wallis test by ranks, with post hoc Dunn’s test where appropriate. To measure the specificity and sensitivity of the p-tau181 test, we calculated the area under the curve (AUC) of the receiver operating characteristics (ROC) using the ‘AUC’ package for R. A repeated measures one-way ANOVA with a Tukey’s post hoc test, adjusted for age, was used to demonstrate the trajectories of p-tau181 over time.

## Results

### Participant characteristics and the effect of age and sex on plasma p-tau

One hundred and fifteen participants were included in this study (111 included at timepoint 1 and four additional participants added at timepoint 2). Table 1 describes the demographical characteristics of the cohort categorised by clinical diagnosis and then by neuropathological diagnosis.

**Table 1:** Demographics of the 115 participants included in this study categorised by the clinical diagnosis assigned at each plasma collection timepoint and then neuropathological diagnosis given at post-mortem

	Timepoint 1				Timepoint 2					Timepoint 3			
Years to post-mortem*,* mean (range)	7.9 (6.33–9.4)				4.2 (2.9–6.1)					2.1 (0.9–3.2)			
Clinical diagnosis	All (*n* = 111)	CU (*n* = 28)	MCI (*n* = 6)	AD dementia (n = 77)	All (*n* = 100)	CU (*n* = 24)	MCI (*n* = 5)	AD dementia (*n* = 71)		All (*n* = 87)	CU (*n* = 20)	MCI (*n* = 5)	AD dementia (*n* = 62)
Number of participants	111	28	6	77	100	24	5	71		87	20	5	62
Age, mean years (SD)	82.0 (7.1)	82.2 (6.5)	87.1 (6.1)	81.7 (7.6)	87.0 (6.9)	86.2 (7.3)	90.2 (6.0)	84.7 (7.4)		89.1 (7.0)	88.4 (7.1)	91.6 (6.5)	87.1 (7.1)
Sex, *n* (% females)	64 (57.5)	18 (64.3)	4 (66.7)	42 (53.9)	60 (60.0)	15 (62.5)	3 (60.)	42 (59.2)		53 (60.9)	13 (65.0)	4 (0.8)	36 (58.1)
MMSE score, mean (SD)	17.7 (10.5)	29.2* (0.91)	26.3* (1.33)	12.3 (8.9)	15.8 (11.0)	28.3* (1.2)	26.0* (1.5)	10.5 (6.7)		14.9 (11.3)	28.7* (1.3)	22.0* (3.5)	8.7 (7.5)
p-tau181, mean pg/mL (SD)	25.6 (11.3)	19.3* (9.9)	26.7 (11.0)	28.4 (9.6)	29.4 (11.8)	20.7* (6.7)	31.7 (18.8)	34.1 (11.1)		29.3 (10.7)	21.7* (12.2)	28.7 (6.3)	31.8 (10.2)

	Timepoint 1				Timepoint 2					Timepoint 3			
Years to post-mortem*,* mean (range)	7.9 (6.33–9.4)				4.2 (2.9–6.1)					2.1 (0.9–3.2)			
Neuropathological diagnosis	All (*n* = 111)	Control (*n* = 11)	Non-AD (*n* = 33)	AD (*n* = 67)	All (*n* = 100)	Control (*n* = 9)	Non-AD (*n* = 28)	AD (*n* = 63)		All (*n* = 87)	Control (*n* = 8)	Non-AD (*n* = 23)	AD (*n* = 56)
Age, mean (SD)	82.0 (7.1)	83.9 (5.5)	82.0 (7.1)	81.8 (7.6)	87.0 (6.9)	86.0	82.5	84.1		89.1 (7.0)	89.2	86.4	84.4
Sex (% females)	64 (57.5)	8 (61.5)	18 (54.5)	38 (56.7)	60 (60.0)	6 (66.7)	16 (57.1)	38 (60.3)		53 (60.9)	5 (62.5)	13 (56.4)	35 (62.5)
MMSE score, mean (SD)	17.7 (10.5)	28.8*^+^ (1.2)	19.1 (7.2)	15.2 (9.9)	15.8 (11.0)	28.6*^+^ (1.1)	16.9 (6.7)	13.3 (8.7)		14.9 (11.3)	28.7* (0.8)	15.6* (6.3)	8.8 (13.4)
p-tau181, mean pg/mL (SD)	25.6 (11.3)	18.1* (5.7)	17.3* (4.0)	30.5 (11.6)	29.4 (11.8)	18.7* (6.1)	19.5* (3.2)	35.4 (12.5)		29.3 (10.7)	20.4* (2.5)	20.9* (4.1)	34.0 (10.3)

NB: AD neuropathology group includes AD with no reported co-pathology (*n* = 25), AD plus cerebral amyloid angiopathy (*n* = 21), AD plus Lewy body pathology (LBD, *n* = 10), AD plus TDP43 pathology (*n* = 15). The non-AD pathology group includes 4R tauopathies (cortical basal degeneration, *n* = 2, progressive supranuclear palsy *n* = 2, argyrophilic grain disease, *n* = 5), cerebral amyloid angiopathy (*n* = 5), frontotemporal lobe degeneration (*n* = 5), Lewy body dementia (*n* = 7), vascular dementia (*n* = 7). Age and MMSE differences between clinical or neuropathological diagnoses in each timepoint were assessed with a one-way ANOVA followed by a pairwise Tukey corrected pairwise post hoc comparisons. Differences in sex distribution were assessed with a Pearson’s *χ*2 test. Significant differences compared to AD dementia or AD are depicted with an asterisk (*). Significant differences compared to non-AD pathology are depicted with a plus (^**+**^)

We first examined the cohort based on clinical diagnoses assigned by a clinical examination at each timepoint. There were no significant differences in age or sex between the groups at any timepoint throughout the study. As expected, a significant decrease in cognitive performance (MMSE) was observed in AD dementia patients as compared to MCI and CU at all timepoints (*P* < 0.0001). No differences in MMSE were observed between CU and MCI. Next, we assessed the demographic variables based on neuropathological diagnosis. Once more, at each timepoint, the neuropathological groups did not differ significantly regarding age or sex. The AD pathology group had a significantly lower MMSE than controls at each timepoint (timepoint 1, *P* < 0.0001; timepoint 2, *P* = 0.001; timepoint 3, *P* < 0.0001). The non-AD group had significantly lower MMSE than controls at timepoint 1 (*P* = 0.013) and timepoint 2 (*P* = 0.015) but not at timepoint 3. A statistically significant difference in MMSE score between non-AD and AD was only observed at timepoint 3 (*P* < 0.0001).

In the whole cohort, there was significant but weak association between age and plasma p-tau181 at all timepoints (*r* = 0.251–0.302, *P* < 0.050). No differences in sex were observed after a correction for age, although the mean p-tau181 concentration was consistently higher in females at all timepoints.

### Plasma p-tau181 associates with post-mortem-confirmed AD pathology

**Fig. 2:**
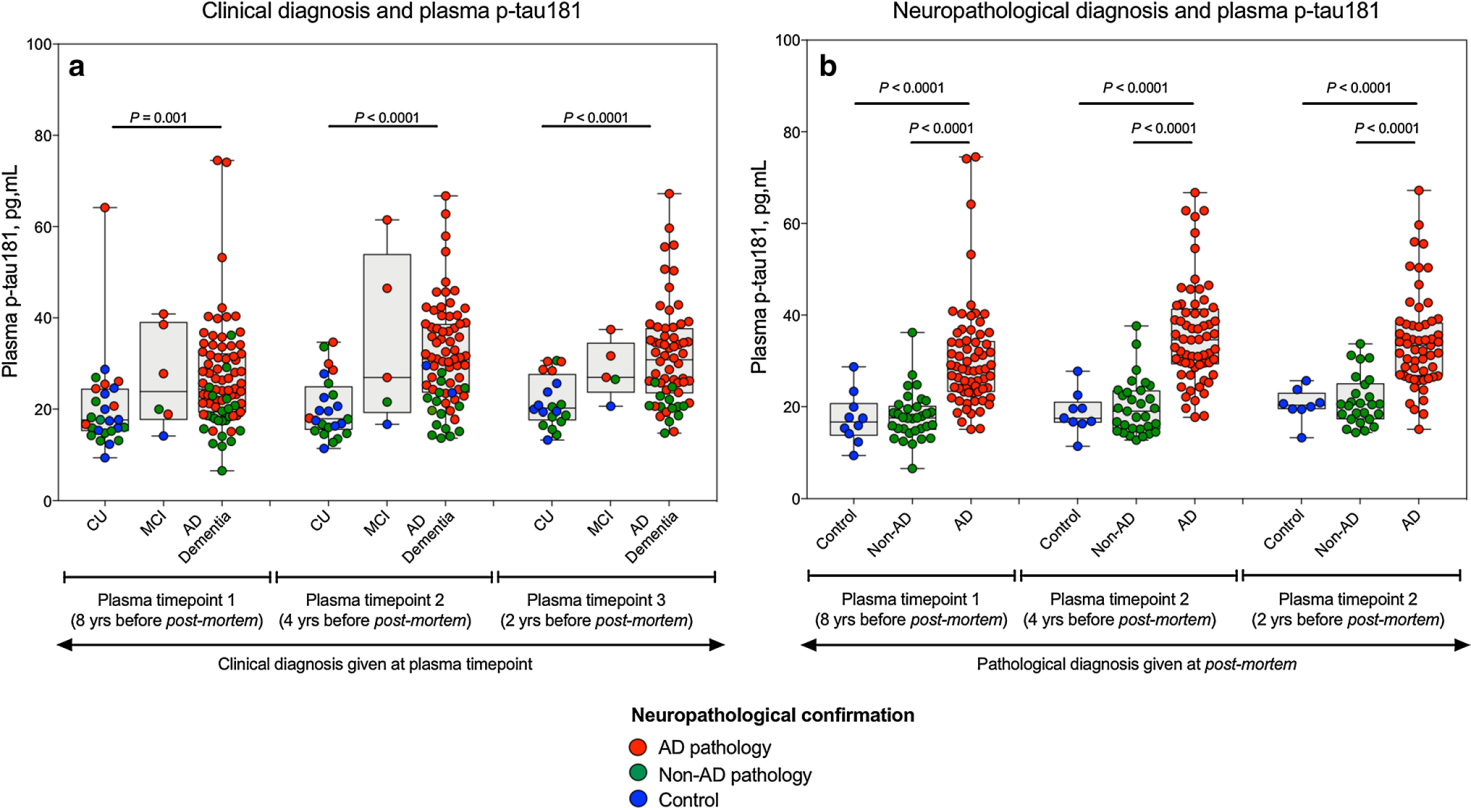
Plasma p-tau181 associates with post-mortem confirmed AD pathology. Plasma concentrations of p-tau181 categorised by clinical diagnosis (**a**) and neuropathological diagnosis given at post-mortem (**b**). The concentrations of plasma p-tau181 are shown at three different time points; 8 years, 4 years prior and 2 years prior to post-mortem. In **a**, the clinical diagnosis assigned at each timepoint are given on the *x*-axis and individual data points are colour coded according the neuropathological diagnosis given at post-mortem to visualise the disparity in clinical classification given at each visitation and underlying pathology uncovered at post-mortem; controls (blue), AD (red), non-AD (green). At timepoint 1, 111 individuals were included (control, *n* = 27; MCI, *n* = 6; AD, *n* = 78). At timepoint 2, 100 individuals were included (control, *n* = 24; MCI, *n* = 5; AD, *n* = 71). At timepoint 3, 87 individuals were included (control, *n* = 20; MCI, *n* = 5; AD, *n* = 62). In **b**, the neuropathological diagnosis given at post-mortem is on the *x*-axis with the same colour coding as (**b**)

We first tested whether plasma p-tau181 differed between clinical syndromes (Fig. 2a). At all three timepoints studied, plasma p-tau181 was significantly higher in AD dementia syndrome as compared to CU individuals (timepoint 1, *P* = 0.001; timepoint 2, *P* < 0.0001; timepoint 3, *P* < 0.0001), but not compared to MCI (Fig. 2a). However, only 75% of the clinically diagnosed AD dementia patients exhibited AD pathology at post-mortem. Therefore, we next compared the levels of plasma p-tau181 between the three neuropathological diagnosis groups (i.e*.*, controls, non-AD and AD; depicted by colour coding in Fig. 2a), regardless of their prior clinical diagnosis. Interestingly, we found that the AD pathology group had significantly higher plasma p-tau181 as compared to the control (*P* < 0.0001) and non-AD pathology groups (*P* < 0.0001, Fig. 2b). This increase occurred at all three plasma timepoints. On the contrary, no differences between pathologically confirmed controls and non-AD pathology were observed at any timepoint (Fig. 2b). Note that the increase in plasma p-tau181 in the AD pathology compared to the control group ranged between 68.5 and 89.3%, a higher increase than that found between the clinical diagnosis of AD dementia syndrome and CU (47.1–59.1%). This favours the idea that plasma p-tau181 reflects AD pathology, regardless of the clinical presentation. The AD pathology group also included individuals with mixed pathology (i.e*.*, with additional Lewy body disease and/or TDP-43 pathology) and excluding them from the analyses did not change the result.

**Fig. 3:**
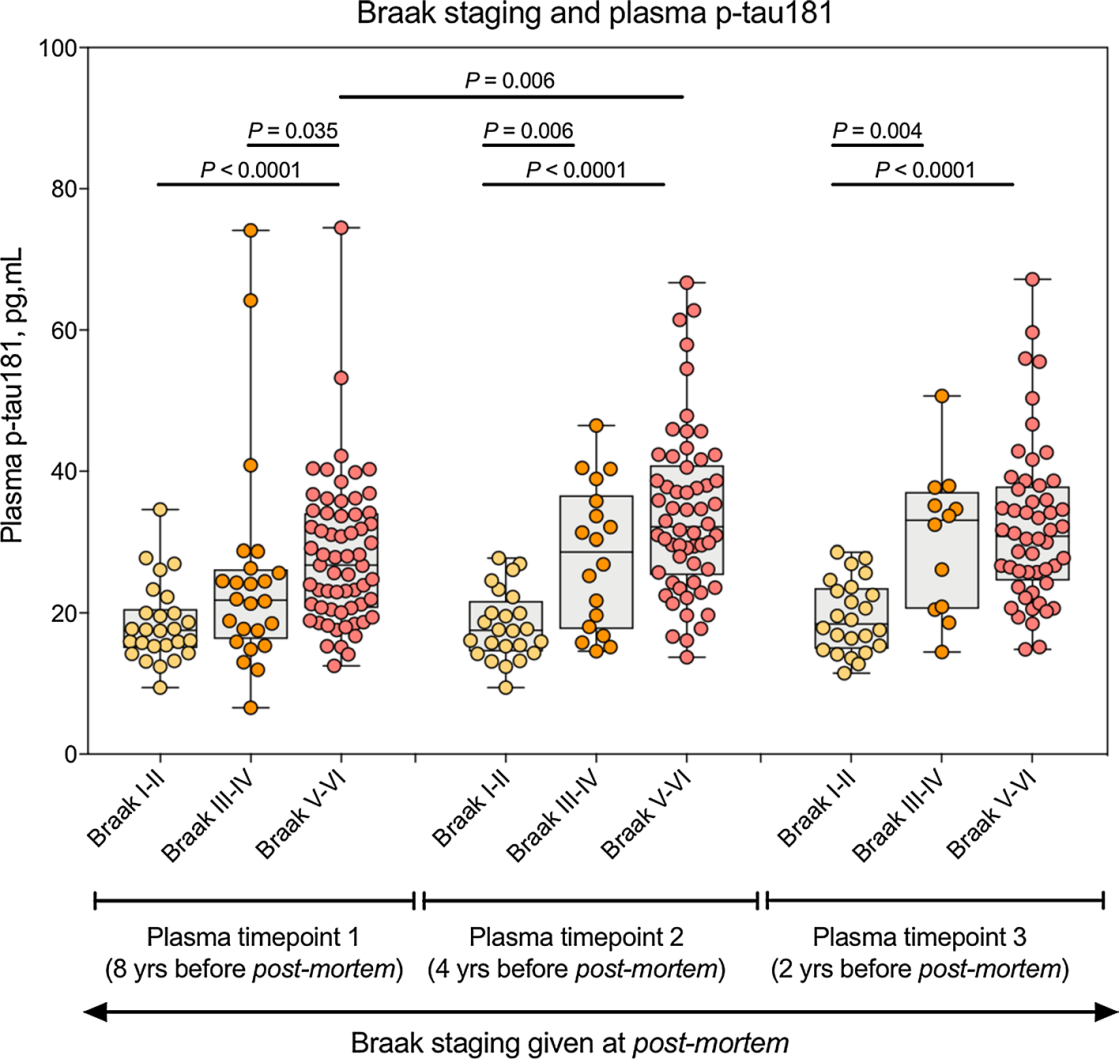
Plasma p-tau181 associates Braak staging. Plasma concentrations of p-tau181 categorised by Braak staging assigned at post-mortem (Braak I–II, Transentorhinal; Braak III–IV, Limbic; Braak V–VI, Isocortical). The concentrations of plasma p-tau181 are shown at three different time points; 8 years, 4 years prior and 2 years prior to post-mortem

We also tested whether plasma p-tau181 changes as a function of Braak stages, which scores the spread of NFT in the brain. Plasma p-tau181 increased in Braak stages V–VI as compared to Braak stages I–II at all timepoints (*P* < 0.0001, Fig. 3). At 8 years from post-mortem, plasma p-tau181 was significantly increased in Braak V–VI as compared to III–IV (*P* = 0.035, Fig. 3) whereas no statistical difference was observed between Braak I–II and Braak III–IV. However, at later timepoints (4 and 2 years before post-mortem), significant increases where only observed between I–II and III–IV (*P* < 0.006) and not between III–IV and V–VI. Together, these results show that plasma p-tau181 is increased in AD pathology, even years before the eventual pathological confirmation.

### Plasma p-tau181 predicts AD pathology from non-AD pathology 8 years before post-mortem irrespective of clinical diagnosis

**Fig. 4:**
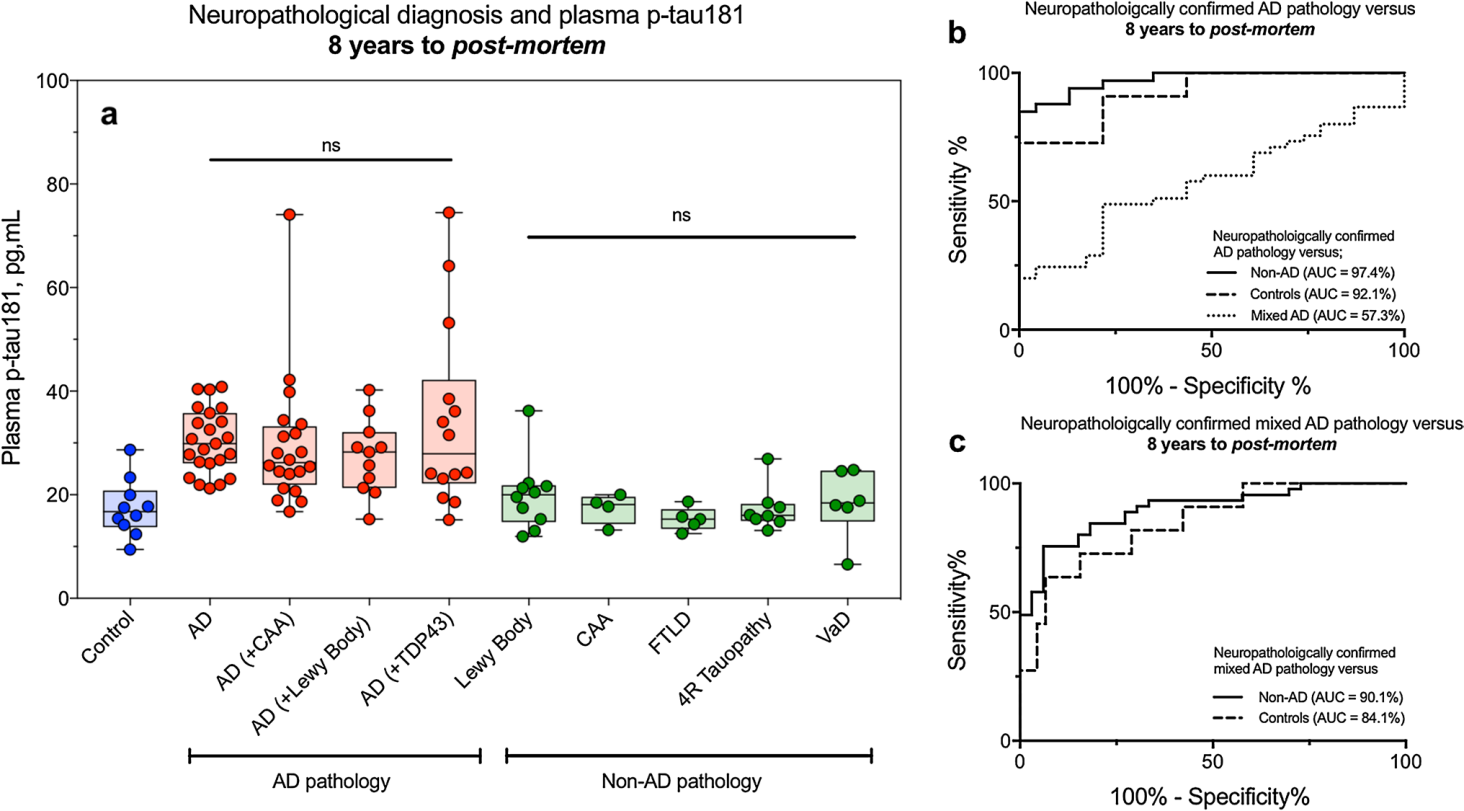
Plasma p-tau181 predicts AD pathology from non-AD pathology 8 years before post-mortem irrespective of clinical diagnosis. **a** A detailed breakdown of neuropathological classification of individuals at post-mortem on the *x*-axis and their corresponding plasma p-tau181 at timepoint 1 (8 years before post-mortem). ROC curves demonstrate the ability of plasma p-tau181 to separate AD (**b**) and mixed AD (**c**) from other neuropathological confirmed pathologies 8 years prior to post-mortem

Figure 4a shows a detailed breakdown of neuropathological classification of individuals at post-mortem on the *x*-axis and their corresponding plasma p-tau181 levels at timepoint 1 (8 years before post-mortem). There was no significant difference in plasma p-tau181 between AD and all AD plus co-pathologies (Fig. 4a) which was also observed at subsequent timepoints (Supplementary Fig. 1a, b, online resource). Additionally, there was no change in p-tau181 between non-AD pathologies at timepoint 1; however, significant increases of p-tau181 were observed in Lewy body dementias as compared to other non-AD dementias at timepoints 2 and 3 (Supplementary Fig. 1a, b, online resource) but not at timepoint 1.

We hypothesised that plasma p-tau181 would discriminate between AD pathology and non-AD pathology several years before the neuropathological confirmation. To test this hypothesis, we conducted a ROC analysis and demonstrated that plasma p-tau181 measured 8 years prior to post-mortem has a high area under the curve (AUC) in separating AD from non-AD pathologies (AUC = 97.4%, 95% CI = 94.1–100%) and controls (AUC = 92.1%, 95% CI = 82.4–100%, Fig. 4b). Similarly, a good performance was demonstrated in separating mixed AD pathologies from non-AD pathologies (AUC = 90.1%, 95% CI = 83.4–96.8%) and controls (AUC = 84.1%, 95% CI = 72.4–97.3%, Fig. 4c) 8 years prior to post-mortem. The low AUC in separating AD from mixed AD pathologies (AUC = 57.3%) demonstrates that plasma p-tau181 is not sensitive to the contribution of co-pathologies.

### Longitudinal trajectories of p-tau181 are dependent on post-mortem pathology

**Fig. 5:**
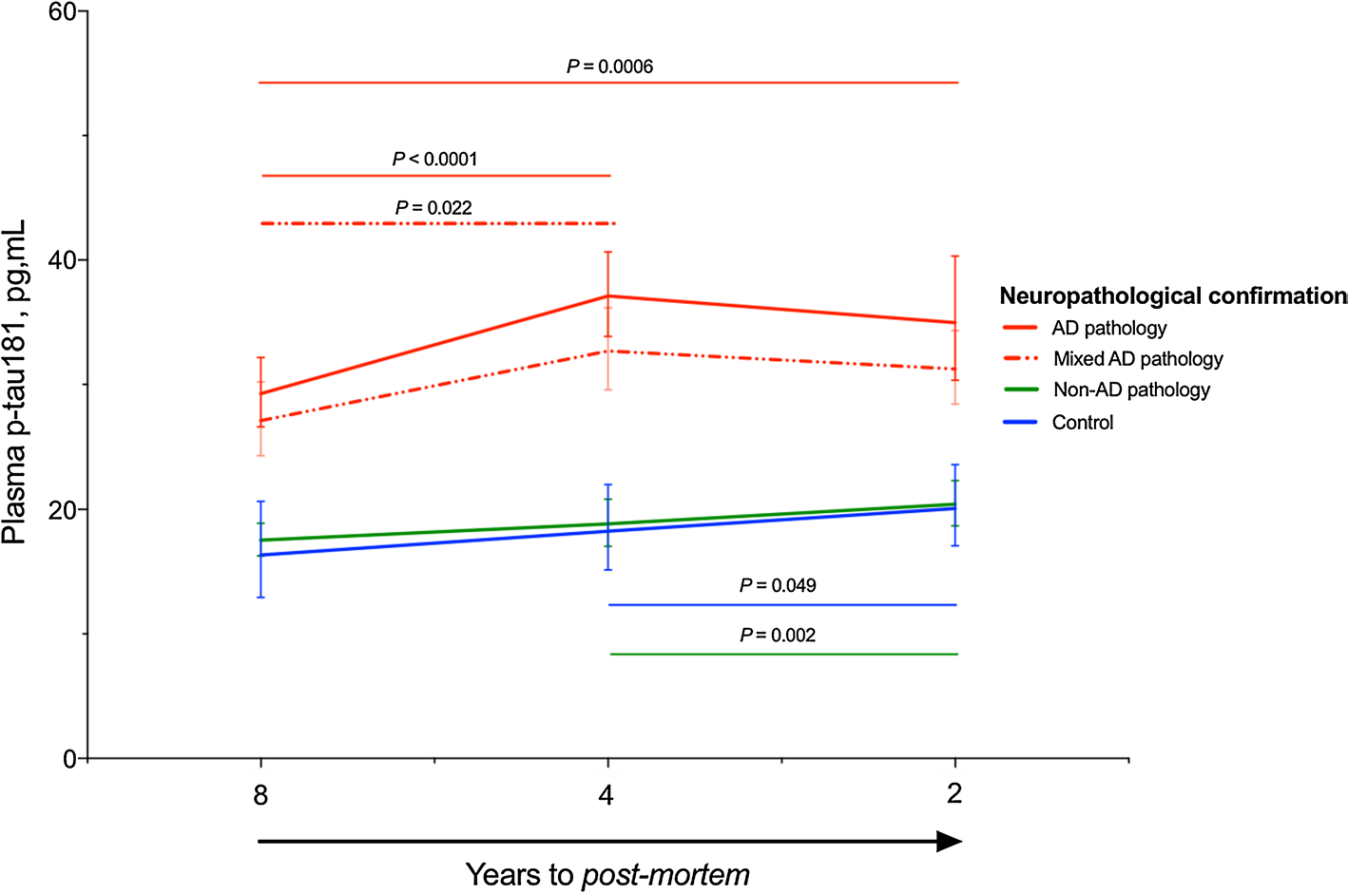
Longitudinal trajectories of p-tau181 are dependent on post-mortem pathology. A repeated measures one-way ANOVA demonstrated the longitudinal changes of plasma p-tau181 in AD, mixed AD, non-AD and controls confirmed at post-mortem

Finally, we assessed whether levels of plasma p-tau181 longitudinally changes in patients eventually diagnosed with AD pathology. Patients were categorised based on post-mortem classification and only individuals with ≥ 2 timepoints were included, this resulted in 100 individuals (controls, *n* = 9; AD, *n* = 22; mixed AD, *n* = 41; non-AD, n = 28) being included in the analysis. In a repeated measures one-way ANOVA, we found a statistically significant effect of time on plasma p-tau181 for AD pathology [*F* (1.56, 31.27) = 11.35, *P* < 0.0001], mixed AD pathology [*F* (1.67, 59.41) = 6.24, *P* = 0.005] and non-AD pathology [*F* (1.71, 41.83) = 14.52, *P* < 0.0001]. A Tukey post hoc analysis demonstrated that plasma p-tau181 significantly increased from timepoint 1 to timepoint 2 in both the AD pathology and AD mixed pathology groups (*P* < 0.0001 and *P* = 0.022, respectively). However, the plasma p-tau181 increase in the AD group was more pronounced than that of the AD mixed pathology group. Plasma p-tau181 was only significantly increased from timepoint 1 to timepoint 3 in the AD pathology group (*P* = 0.0006), although an increase approaching significance was also observed in the mixed AD pathology group (*P* = 0.060, Fig. 5). Interestingly, while no significant change in plasma p-tau181 concentration was observed between timepoints 2 and 3 for the AD pathology or AD mixed pathology groups, a mild but significant increase was observed for both the control (*P* = 0.049) and non-AD pathology groups (*P* = 0.002). Together, these results suggest that individuals who develop AD pathology undergo an increase of plasma p-tau181 several years before the final neuropathological diagnosis. Interestingly, Lewy body dementias demonstrated a similar pattern to AD and mixed AD pathologies where an approaching significant increase (*P* = 0.065) was observed between timepoints 1 and 2, which plateaued at timepoint 3 (Supplementary Fig. 2, online resource).

## Discussion

In a longitudinal cohort with neuropathological characterisation, we demonstrated that plasma p-tau181 predicts AD pathology at least 8 years prior to death and neuropathological confirmation, and accurately discriminates between AD and non-AD pathologies. Our data suggest, an individual clinically diagnosed of AD dementia syndrome but with low concentrations of plasma p-tau181 is more indicative of non-AD than AD pathology. We have also demonstrated that plasma p-tau181 increases over time in cases with AD pathology, most likely in parallel to neurofibrillary tangle neurodegeneration (as shown by Braak stages). This increase, however, plateaus at the very advanced stages of the disease. Altogether, our results support the idea of using plasma p-tau181 as a biomarker of AD at the clinical setting or in clinical trials when CSF and/or PET biomarkers are not available. Alternatively, plasma p-tau181 could be used as a pre-screening tool to select those patients who would further undergo lumbar puncture or PET imaging.

As the field of fluid biomarkers in neurodegeneration moves towards targeted analysis in blood, several novel assays have been developed. Assays targeting Aβ [22, 23, 28], t-tau [8, 24] and NfL [2, 5, 18] in blood have demonstrated promising results. However, they are compromised by either substantial peripheral expression of the targeted protein [26], poor correlations with CSF measures of the same protein [19] and large overlaps between the neurodegenerative disease groups [1, 10]. These limitations may make the use of these biomarkers to diagnose and/or predict the development of AD pathology more difficult at the individual level. A major breakthrough has been the development of assays to sensitively measure plasma p-tau181 by us and other groups [15, 16, 32]. In contrast to the aforementioned biomarkers, p-tau181 can be robustly measured in plasma, is highly specific for AD, provides high diagnostic accuracy for discriminating AD and non-AD dementia, and it also finely discriminates between Aβ-positive CU older adults from those that are Aβ-negative. Furthermore, plasma p-tau181 correlates with CSF p-tau181 and identifies tau PET uptake, suggesting that p-tau181 found in plasma is predominately derived from the central nervous system and not from a peripheral source.

To the best of our knowledge, this is the first study of longitudinal plasma p-tau181 with a confirmed neuropathological diagnosis. Although the clinical diagnosis of AD by an expert neurologist is very reliable, it is not rare to find discordances between the clinical and the final pathological diagnosis [4]. In our study, we initially demonstrated a significant increase in plasma p-tau181 in the AD dementia syndrome group compared to the CU groups. However, there was a large overlap in plasma p-tau181 with the control group likely owing to the lack of CSF and PET characterisation in this cohort. Remarkably, when this comparison was performed between pathologically defined groups, the magnitude of the differences between the AD pathology and control group was considerably higher. These results indicate that plasma p-tau181 is specific for AD pathology, irrespective of whether the clinical presentation resembles a typical AD dementia or another type of dementia. We further confirmed this idea by the fact that plasma p-tau181 discriminates AD pathology from non-AD pathology with an AUC of 97.4% 8 years prior to post-mortem, which is of equivalent performance to the well-established CSF AD core biomarkers (Aβ42, p-tau and t-tau) or Aβ and Tau PET. Our study to some extent mimics a still common situation in several non-specialised clinics, where these CSF or PET biomarkers are not available. Our results open the possibility of routinely using plasma p-tau181 to improve the confidence in administering symptomatic treatment (e.g. acetylcholinesterase inhibitors or memantine), or better inform on patient management. Also, it may be used in both clinical practice and clinical trials as a first screening tool that may be followed, if needed, by CSF or PET biomarker confirmation.

In addition to comparing plasma p-tau181 in clinical and neuropathological classifications, we also examined the relationship of plasma p-tau181 with Braak staging. We observed that a significant increase of plasma p-tau181 occurred between Braak I–II (Transentorhinal) and V–VI (Isocortical) at all timepoints. We also observed, at 8 years prior to post-mortem, a significant increase of plasma p-tau181 between Braak stages III–IV (Limbic) and V–VI but this was not apparent at later timepoints. Instead, 4 years and 2 years antemortem, the significant differences occurred between Braak stages I–II and III–IV. Moreover, plasma p-tau181 concentrations in individuals classified as Braak stage III–IV followed an increasing mean trend across all three timepoints. In contrast, no mean change in Braak stages I–II was observed and individuals that reached a higher degree of tau pathology, that is Isocortical Braak V–VI, began to plateau after initial significant increase between 8 and 4 years antemortem. These trajectories are probably parallel to that of Aβ pathology, which also plateaus at more advanced stages. In fact, plasma p-tau181 highly correlates with Aβ PET [15, 16, 32]. These results suggest that plasma p-tau181 is a good diagnostic marker in both early and late stages of the disease. However, plasma p-tau181 may only be useful as a biomarker of the burden of the disease at early stages, when its levels follow an increasing trajectory, rather than at late stages, when the levels plateau.

Another novel contribution of our study is that we demonstrate that the trajectories of plasma p-tau181 change over several years in patients with AD. The availability of repeated plasma samples over the course of a decade allowed us to define this trajectory. Interestingly, we found that the main increase in plasma p-tau181 in the AD pathology group occurred from timepoint 1 (8 years prior to post-mortem) and timepoint 2 (4 years prior to post-mortem). However, between timepoint 2 (4 years prior to post-mortem) and timepoint 3 (2 years prior to post-mortem) p-tau181 began to plateau. This suggests that the main increase in plasma p-tau181 occurs several years before there is overt AD pathology. In the case of AD with contaminate pathology, this increase was less steep but still significant and following the same trajectory as AD pathology. These findings are consistent with the longitudinal studies showing that CSF p-tau or t-tau does not increase, or it may even decrease, in advanced stages of the disease, which may indicate a deceleration in neurodegeneration due to substantial neuronal loss [31]. This hypothesis has also gained support from recent stable isotope labelling kinetics (SILK) studies that track the turnover of tau in the human central nervous system (CNS) [27]. In contrast to the AD pathology groups, we observed a slight but statistically significant increase of plasma p-tau181 between timepoints 2 and 3 for the non-AD dementia and control groups. This slight increase cannot be attributed to underlying Aβ pathology, due to the neuropathological report, but there may be other factors that have an effect on p-tau181, such as ageing or co-pathology (TDP-43, α-synuclein).

Our study has some limitations. First, the availability of CSF or PET biomarker data would have allowed us to compare the predictive value of plasma p-tau181 with accepted gold-standard biomarkers for AD. Second, the number of CU participants that eventually were pathologically diagnosed as AD was low and, therefore, we could not test whether plasma p-tau181 can also predict AD pathology at early preclinical stages. In a similar manner, the number of MCI patients in this study was low and, therefore, the progression from MCI to AD dementia could not be investigated. Furthermore, the AD dementia patients were quite impaired already at the first timepoint, with an average MMSE score of 12. Finally, detailed Aβ pathological data, such as Thal staging, were not available for all cases. However, recently, Thal staging has been shown to not substantially contribute to predicting antemortem cognition as compared to neuritic plaque scores and Braak NFT stages [30]. The main strength is that we studied a very well-characterised cohort of participants, with longitudinal samples and neuropathological confirmation. Furthermore, we used a very sensitive and robust p-tau181 assay which, importantly, can be easily set up in other centres and hence replicate our results.

In conclusion, our study demonstrates that plasma p-tau181 predicts AD pathology, even if the blood sample was obtained several years before the post-mortem examination. This has obvious consequences in both the design of clinical trials and at the routine clinical practice. Plasma p-tau181 could be used as a rapid and cost-effective screening tool for participant selection for therapeutic trials of AD. Furthermore, the high accuracy of p-tau181 in predicting confirmed AD neuropathology may guide clinicians in an accurate diagnosis of the underlying mechanism causing cognitive decline (AD pathology, non-AD or mixed) and, therefore, symptomatic treatment and patient management can be governed at the earliest stage with a higher degree of confidence.

## Electronic supplementary material

Below is the link to the electronic supplementary material.

Supplementary file1 Supplementary Figure 1. Plasma p-tau181 increases in AD pathology 4 years and 2 years before post-mortem. The detailed breakdown of neuropathological classification of individuals at post-mortem on the x-axis and their corresponding plasma p-tau181 at 4 years (Supplementary Fig. 1a) and 2 years (Supplementary Fig. 1b) before post-mortem. (TIFF 6078 kb)

Supplementary file2 Supplementary Figure 2. Plasma p-tau181 levels in Lewy body pathology 8 years, 4 years and 2 years before post-mortem confirmation (TIFF 6078 kb)
