# Supplementary figures and images for: Plasma p-tau181 accurately predicts Alzheimer’s disease pathology at least 8 years prior to post-mortem and improves the clinical characterisation of cognitive decline
Source: Acta Neuropathol. 2020 Jul 27;140(3):267–78. doi: 10.1007/s00401-020-02195-x (PMC7423866; doi:10.1007/s00401-020-02195-x)

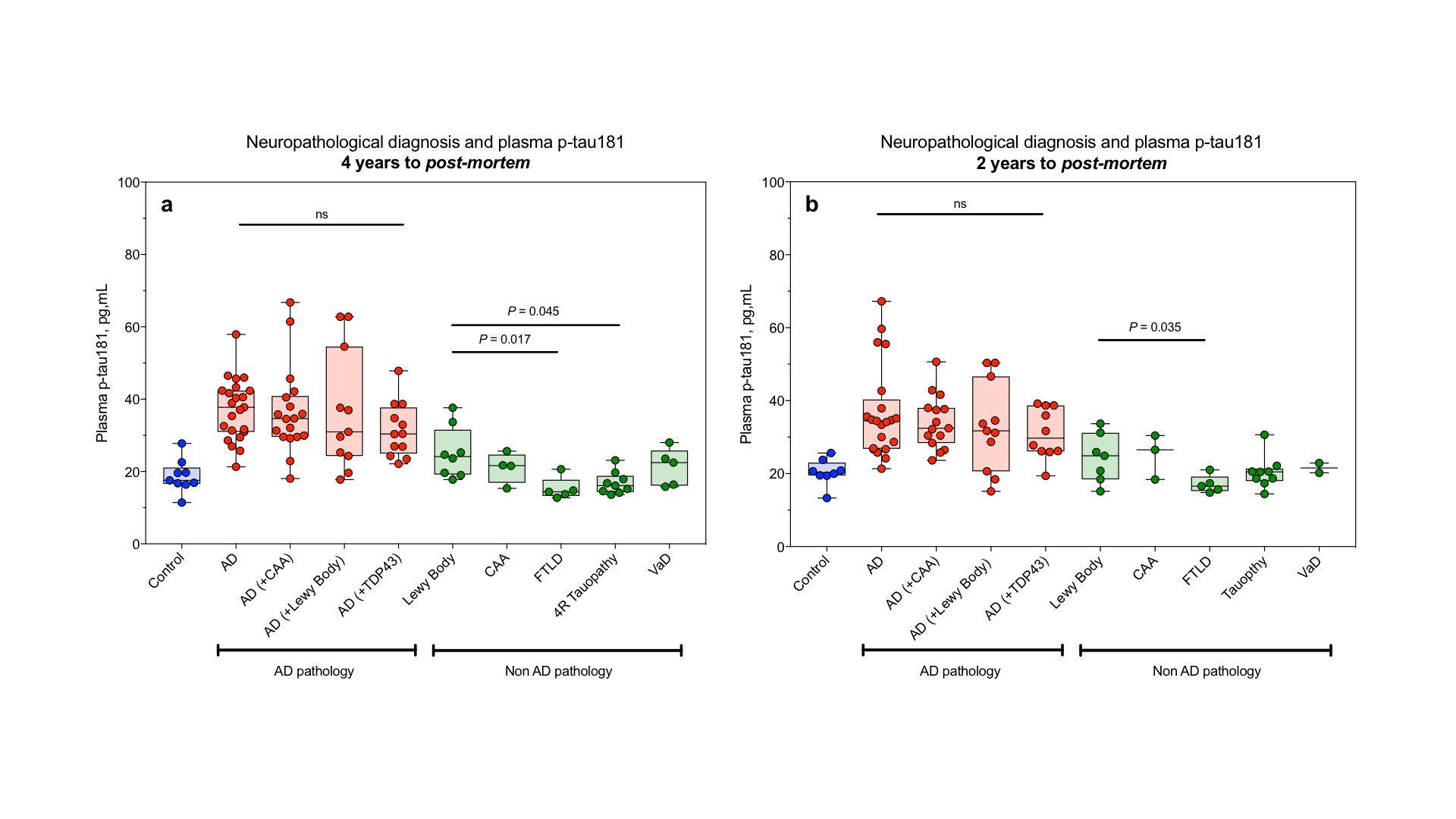

Supplement: Supplementary file 1 — Supplementary file1 Supplementary Figure 1. Plasma p-tau181 increases in AD pathology 4 years and 2 years before post-mortem. The detailed breakdown of neuropathological classification of individuals at post-mortem on the x-axis and their corresponding plasma p-tau181 at 4 years (Supplementary Fig. 1a) and 2 years (Supplementary Fig. 1b) before post-mortem. (TIFF 6078 kb) [file 401_2020_2195_MOESM1_ESM.tiff]

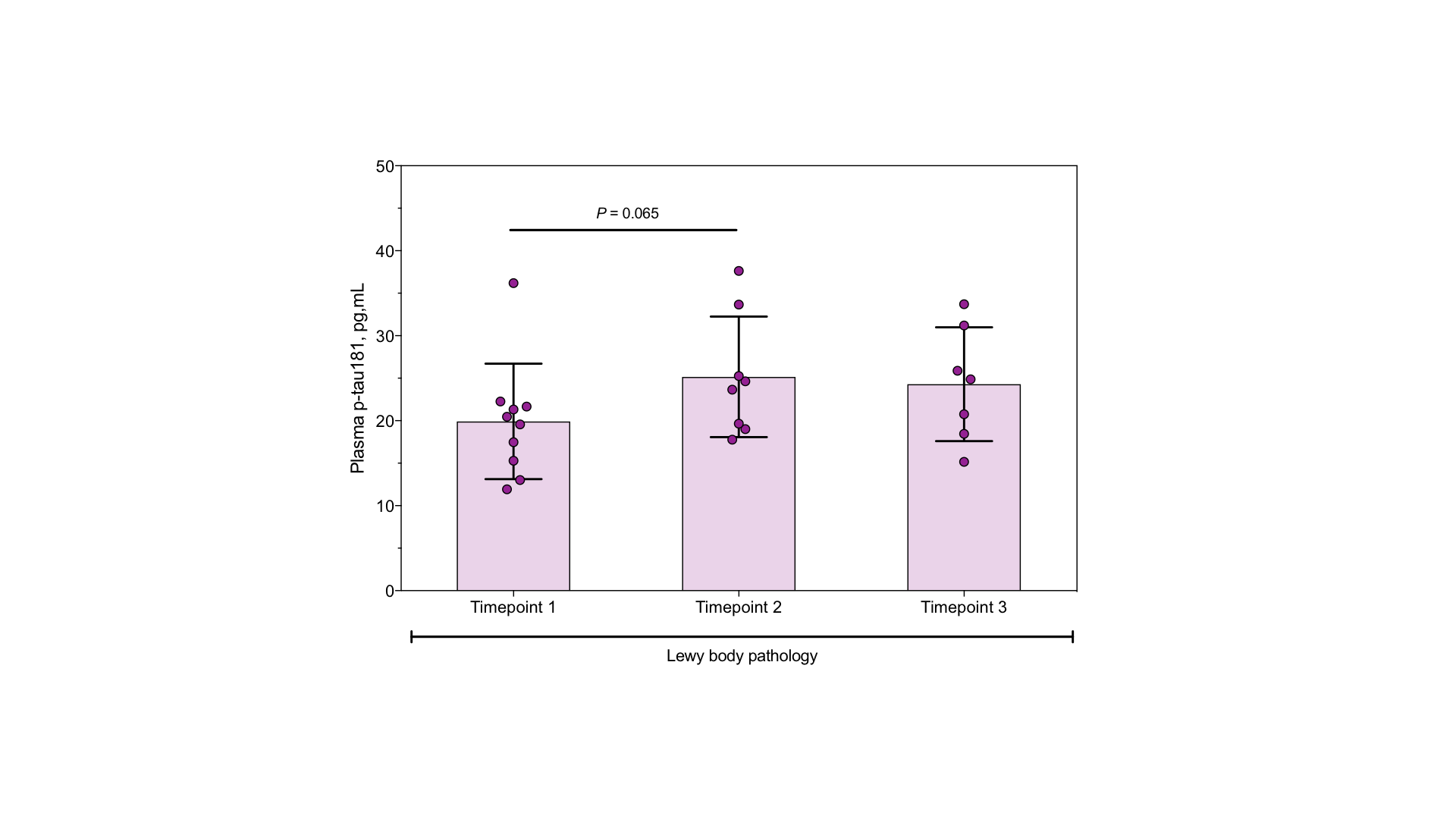

Supplement: Supplementary file 2 — Supplementary file2 Supplementary Figure 2. Plasma p-tau181 levels in Lewy body pathology 8 years, 4 years and 2 years before post-mortem confirmation (TIFF 6078 kb) [file 401_2020_2195_MOESM2_ESM.tiff]
